# Eye Tracking Supported Human Factors Testing Improving Patient Training

**DOI:** 10.1007/s10916-021-01729-4

**Published:** 2021-03-25

**Authors:** Kerrin Elisabeth Weiss, Christoph Hoermandinger, Marcus Mueller, Marianne Schmid Daners, Evgenij V. Potapov, Volkmar Falk, Mirko Meboldt, Quentin Lohmeyer

**Affiliations:** 1 Product Development Group Zurich, Department of Mechanical and Process Engineering, ETH Zurich, Leonhardstrasse 21, 8092 Zürich, Switzerland; 2 Department of Cardiothoracic and Vascular Surgery, German Heart Center Berlin, Berlin, Germany; 3 DZHK (German Centre for Cardiovascular Research), Partner Site Berlin, Berlin, Germany; 4 Department of Cardiovascular Surgery, Charité – Universitätsmedizin Berlin, Berlin, Germany; 5 Department of Health Sciences and Technology, ETH Zurich, Zurich, Switzerland

**Keywords:** Eye tracking, Training, Emergency scenario, Mechanical circulatory support, Usability, Pump-off time

## Abstract

The handling of left ventricular assist devices (LVADs) can be challenging for patients and requires appropriate training. The devices’ usability impacts patients’ safety and quality of life. In this study, an eye tracking supported human factors testing was performed to reveal problems during use and test the trainings’ effectiveness. In total 32 HeartWare HVAD patients (including 6 pre-VAD patients) and 3 technical experts as control group performed a battery change (BC) and a controller change (CC) as an everyday and emergency scenario on a training device. By tracking the patients’ gaze point, task duration and pump-off time were evaluated. Patients with LVAD support ≥1 year showed significantly shorter BC task duration than patients with LVAD support <1 year (*p* = 0.008). In contrast their CC task duration (*p* = 0.002) and pump-off times (median = 12.35 s) were higher than for LVAD support patients <1 year (median = 5.3 s) with *p* = 0.001. The shorter BC task duration for patients with LVAD support ≥1 year indicate that with time patients establish routines and gain confidence using their device. The opposite effect was found for CC task duration and pump-off times. This implies the need for intermittent re-training of less frequent tasks to increase patients’ safety.

**Supplementary Information:**

The online version contains supplementary material available at 10.1007/s10916-021-01729-4.

## Introduction

This study investigates the human factors of the HeartWare HVAD (Medtronic, Minneapolis, MN, USA) left ventricular assist device (LVAD) system using eye tracking.

Due to the shortage of donor hearts, LVADs are a common solution for patients with heart failure [1, 2]. The HVAD is one of the commercially available continuous flow LVADs with more than 16,000 implantations worldwide [3, 4]. Although medical complications of HVADs are researched extensively, [5–8] less attention is given to the usability of its battery and controller system including malfunctions [9–11]. The human factors of LVAD wearable components remain a crucial challenge [12, 13].

Because LVADs are critical life-support systems, the device should be self-explanatory and optimization of usability, including intuitive handling and enhanced device-user communication is needed to increase the safety and quality of life [14]. A broad systematic review by Dunn et al. [11] showed existing human factor issues and how they influence user experience. However, an objective and quantitative assessment of human factors of LVAD systems without the use of questionnaires is still an ongoing challenge.

**Fig. 1:**
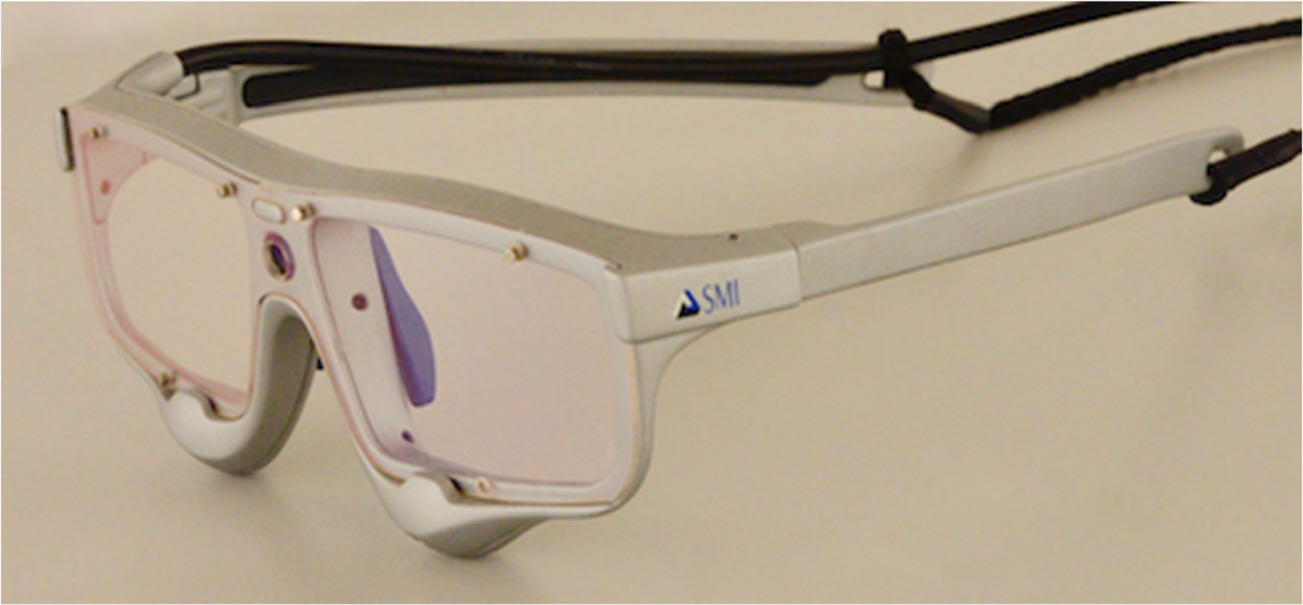
SMI ETG 2 Wireless mobile eye tracking glasses (Senso Motoric Instruments, Teltow, Germany)

**Fig. 2:**
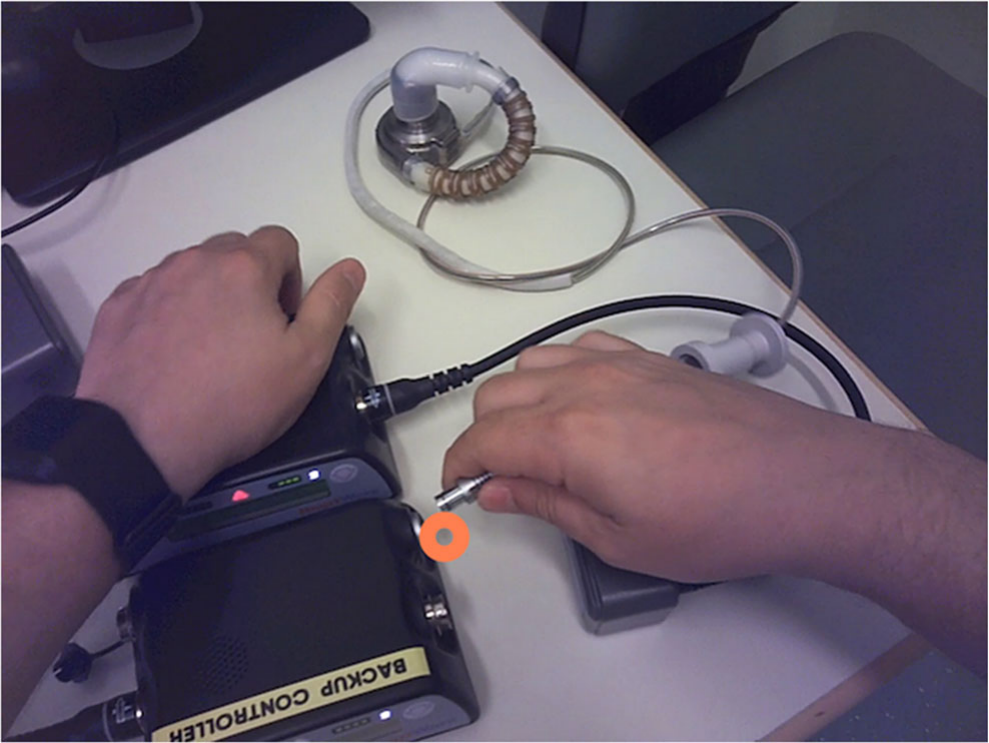
Gaze point of patient during controller change task

A useful tool to overcome this challenge is eye tracking, which enables to uncover common problems during the use of medical devices [15], provides valuable objective insights for user interface design [16] and allows to assess the effectiveness of trainings [17]. It proves as a successful skill and training assessment tool, [18] is applicable in medical education, [19] and allows for comparison between expert and novice surgeons [20–22]. Eye tracking glasses (see Fig. 1) do not restrict the participant in movement, thus allowing them to behave naturally. From the collected data the gaze point of the participant can be computed and displayed in the first person’s view video documentation (“scan path”, see Fig. 2). Furthermore, a comparison between different object and interest points can be investigated by the definition of areas of interest (AOIs). Using quantitative specific AOI measures like dwell time, the total time spend looking at an AOI, allows to draw conclusions regarding the visual behavior and attention.

## Materials and methods

Out of the 240 patients currently supported by HVAD systems at this institution (out of a total of 350 long-term LVAD patients), 44 patients were enrolled in the study after informed consent and 32 patients were included in the analysis. Eight patients (six for analysis) were pre-operative patients, that serve as “novice” control group, while additional three experts (VAD-technicians or perfusionists) formed an expert control group. Patients had to be ≥18 years old, able to consent and speak German (B2). Twelve patients (including two pre-operative patients) had to be excluded from the analysis: three were deficient in reading German, one performed an early start of the controller change, for two patients the study had to be aborted due to an adverse emotional reaction to the controllers’ alarm, technical difficulties arose with three patients and three patients failed to successfully perform the controller change task. The characteristics of participants are shown in Table 1. The patients were stratified based on the duration of LVAD support. The cut-off was defined for the duration of LVAD support of ≥1 year based on the impact of LVAD implantation on patients’ cognitive function [23].

**Table 1:** Patient and expert characteristics. The patient characteristics include the group size (N), the mean (± SD) age in years, the male sex ratio in percent and the mean (± SD) of the duration of LVAD support in days

	Pre patients	Patients LVAD support <1 year	Patients LVAD support ≥1 year	Experts
N	6	14	12	3
Age (years)	57.3 ± 9.9	54.6 ± 14.2	57 ± 14.8	40.3 ± 10.8
Male sex (%)	100	86	92	100
Duration of LVAD support (days)	–	147.1 ± 121.5	1528.4 ± 892.9	–

The standardized institutional training protocol is adapted to the mental ability of the individual patient. Hence the amount of training time depends on numerous variables. Patients have to pass defined criteria to successfully complete their training. This includes the ability of performing multiple battery changes (minimum of eight) and controller changes (minimum of three) with confidence and without any technical mistakes. The training is repeated as many times as needed till the criteria are fulfilled, otherwise a care giver or other means of continuous supervision must be guaranteed before discharge. All patients, except for pre patients, completed their training before taking part in this study. The pre patients were introduced to the HVAD systems right before the study, the components and their handling were explained.

The study was performed at the German Heart Center Berlin and the Paulinenkrankenhaus Berlin in closed rooms with constant conditions. After the participants read the instruction paper, they had to first perform a battery change (BC, everyday scenario) followed by a controller change (CC, rare emergency scenario) using a training HVAD unit. The start signal for the CC was an alarm activated by the study conductor using a separate device. The trained procedure for a controller change is as follows: switch alarm plug from backup controller to malfunctioning controller, switch first battery to backup controller, switch driveline to backup controller, switch second battery to backup controller. After each task the short stress state questionnaire was filled out [24, 25]. Patients not performing the two tasks correctly were directly trained after the study. Video data of the battery and controller change including the gaze point for patients and experts is accessible as supplementary material (see Video 1).

The SMI ETG 2 Wireless mobile eye tracking glasses (Senso Motoric Instruments, Teltow, Germany) with a sampling rate of 60 Hz and a gaze tracking accuracy of 0.5° were used to record the data (see Fig. 1). The system records the scene, including audio, as well as the eyes of the participant to allow the calculation of the participant’s gaze point. Using the gaze point the scan path can be observed displaying where the attention of the participant lies frame by frame.

The task durations of BC and CC were measured. The gaze point was used to find the start and end instants, which are defined as the first time the patients take their gaze from the instruction paper to the HVAD set up and the moment the patients look back at the instruction paper after task completion to turn the page. Furthermore, the pump-off time was measured, which states how long the pump is completely turned off during the CC. The pump is turned off as soon as the driveline is disconnected or if both batteries are disconnected from the malfunctioning controller, the pump is turned on again as soon as it is connected to the backup controller and if it provides power through a connected battery.

Dwell times (DTs) for AOIs of the relevant and irrelevant controller during the CC were calculated. The relevant controller is the one the pump is connected to, while the irrelevant controller is the one without pump. This stimulus switches as soon as the pump is disconnected from the malfunctioning controller and connected to the backup controller. Using the pump-off time, 10 s were used as a comparison criterion [26]. Data analysis was performed with SMI BeGaze 3.6 (Senso Motoric Instruments, Teltow, Germany).

A Pearson Chi-Square test was performed to evaluate gender effects. Differences between age, BC task duration, CC task duration and pump-off time, comparing pre patients, patients with LVAD support <1 year, patients with LVAD support ≥1 year and experts, were analyzed by a Kruskal-Wallis test. The same test was used for DT on irrelevant controller and DT on relevant controller comparing pre patients, LVAD patients with a pump-off time <10 s, LVAD patients with a pump-off time ≥10 s and experts.

It was hypothesized that these groups perform best to worst: Experts, patients ≥1 year, patients <1 year, pre patients.

- H1 (hypothesis 1): Task durations are shorter for patients with a duration of LVAD support ≥1 year compared to patients with LVAD support <1 year.
- H2: The pump-off time is shorter for patients with a duration of LVAD support ≥1 year compared to patients with LVAD support <1 year.
- H3: Patients with a pump-off time ≥10 s have a higher dwell time on the irrelevant controller.

## Results

The groups did not differ by participants gender C^2^(2, *N* = 35) = 1.14, *p* = 0.567 or age C^2^(3, N = 35) = 4.675, *p* = 0.197.

**Fig. 3:**
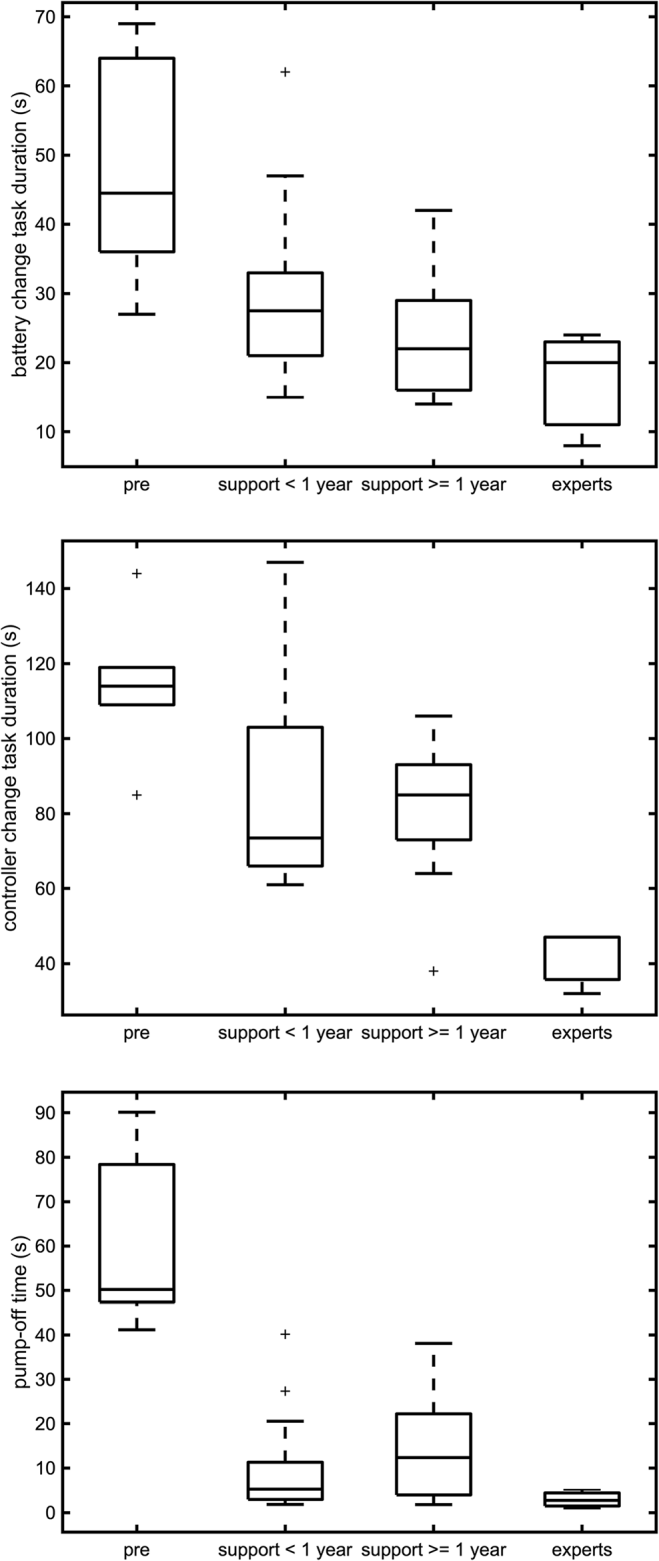
Box plots for battery change and controller change task durations and pump-off time in seconds for pre patients, patients with LVAD support <1 year, patients with LVAD support ≥1 year and experts

The task durations and the pump-off time results are displayed in Table 2 and Fig. 3.

**Table 2:** Battery and controller change task durations and pump-off times. Median (± SD) values and ranges (minima and maxima) of BC task duration, CC task duration and pump-off time in seconds for pre patients, patients with LVAD support <1 year, patients with LVAD support ≥1 year and experts

	Battery change	Controller change	
	Task duration (s)	Task duration (s)	Pump-off time (s)
Pre patients	44.5 ± 16.2	114 ± 19	50.2 ± 19.8
Range	*27–69*	*85–144*	*41.2–90.1*
LVAD support < 1 year	27.5 ± 12.6	73.5 ± 25.6	5.3 ± 11.4
Range	*15–62*	*61–147*	*1.9–40.2*
LVAD support ≥ 1 year	22 ± 8.7	85 ± 18.4	12.4 ± 12.7
Range	*14–42*	*38–106*	*1.8–38.1*
Experts	20 ± 8.3	47 ± 8.7	2.9 ± 2
Range	*8–24*	*32–47*	*1.1–5*
*P*-Values	0.008	0.002	0.001

Patients with LVAD support ≥1 year showed significantly shorter BC task duration than patients with LVAD support <1 year as expected. But their CC task duration were higher than for LVAD support patients <1 year. As expected, the experts have the shortest task duration and the pre patients the longest. This behavior is significant for BC task duration C^2^(3, *N* = 35) = 11.95, *p* = 0.008 and CC task duration C^2^(3, N = 35) = 14.49, *p* = 0.002.

Counter intuitively, the pump-off time for LVAD support patients ≥1 year is higher than for LVAD support patients <1 year. Again, the control groups set the minimal (experts) and maximal (pre patients) pump-off times like expected. The differences between the groups are significant: C^2^(3, *N* = 35) = 17.22, *p* = 0.001.

**Fig. 4:**
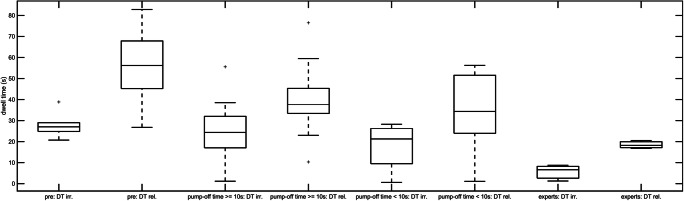
Dwell times in seconds on relevant (rel.) and irrelevant (irr.) controllers for pre patients, patients with LVAD support with a pump-off time ≥10 s, patients with LVAD support with a pump-off time <10 s and experts

Displayed in Table 3 and Fig. 4 are the dwell times (DTs) on the irrelevant and relevant controller.

**Table 3:** Dwell times on relevant and irrelevant controller. Median (± SD) of dwell times (DTs) on relevant (with pump) and irrelevant (without pump) controller in seconds for pre patients, patients with a pump-off time ≥10 s, patients with a pump-off time <10 s and experts

	DT on irrelevant controller (s)	DT on relevant controller (s)
Pre patients	27 ± 6.2	56.2 ± 19.3
Patients with pump-off time ≥ 10 s	24.4 ± 14.4	37.7 ± 16.7
Patients with pump-off time < 10 s	21.3 ± 10.3	34.4 ± 18
Experts	6.6 ± 3.9	18.2 ± 2
P-values	0.03	0.033

For the patients with a pump-off time ≥10s the DTs for relevant and irrelevant controller are higher than for patients with pump-off time <10 s. The differences between the pump-off time <10 s patients and the patients with a pump-off time ≥10 s normed with the DT of patients with a pump-off time ≥10 s are 29.26% for the irrelevant and 14.73% for the relevant controller. The DT on irrelevant controller is low as expected for experts and highest for pre patients. In every group, more DT is spent on the relevant controller. The differences show a significance for the DT on the irrelevant controller with C^2^(3, N = 35) = 8.95, *p* = 0.03 and for the relevant controller with C^2^(3, N = 35) = 8.76, *p* = 0.033.

## Discussion

Five patients were excluded from the analysis due to an adverse emotional reaction to the device or because the patient failed to successfully perform the controller change task. Having to exclude 11.4% of the enrolled patients due to human factors related issues, demonstrates the general need for improvement of the design and handling of LVAD devices. The fact, that the BC task duration is shorter for patients with LVAD support ≥1 year (H1) implies that patients establish a routine with their device. But the opposite effect was found for CC task duration, it is higher for patients with LVAD support ≥1 year (H1). This shows that patients might have lost skills either because they were not trained enough, before their discharge from hospital, or because their training happened too long ago. Both aspects result in a need for adaption of the training processes.

Opposing the expectations of H2, the results show that the average pump-off time is shorter for patients with LVAD support <1 year compared to patients with LVAD support ≥1 year. The difference in quartiles might suggest that the differences between patients’ performances diverge with the increasing time gap to their training. It is suggested that a yearly training of the controller change should become mandatory to increase patients’ safety. That the pump-off time upper ranges are alarmingly high underlines the relevance of training patients to minimize their pump-off time. By analyzing the scan paths, it was discovered that the deviation from the trained order of controller change led to long pump-off times for some participants. This deviation in order was that they first disconnected both batteries instead of only changing one followed by the pump. Not only an increased regularity of CC training, but also the awareness of the pump-off time and its implication should help to prevent this deviation.

As expected for the control groups, the pre patients had the highest BC and CC task durations, pump-off time and dwell times, while the expert had the lowest. That LVAD supported patients were always performing better than pre patients, allows the conclusion that the provided training was effective. But the fact that experts are still able to perform better than LVAD supported patients shows the possibility for further improvement of the training. Regarding H3, i.e. patients with a pump-off time ≥10 s have a higher dwell time on the irrelevant controller than on the relevant controller, no significant results were found. Although the dwell time on the irrelevant controller is smaller than on the relevant controller for every group, the attention spent on the irrelevant controller is too high. The results for the expert group show an approximate ratio of 1:3 between the DT on the irrelevant versus the relevant controller, demonstrating the amount of attention needed for the irrelevant controller.

Contemplating limitations, the criteria of 10 s to separate patients through their pump-off time does not allow conclusions on the physical state of the patients after their pump is off. The patients’ ventricular function strongly influences at which point in time they lose consciousness. Three pre patients had undergone surgery to receive HVAD devices after the study was finished. For one pre patient the decision was made not to implant any LVAD system due to incapability of managing the device. Observations from the study were used in the respective discussions. Although this observation is anecdotal it points to the fact that eye tracking could be used as a tool for patient assessment and to objectively measure their interaction with the system.

Agreeing with Schlögelhofer and Schima’s [13] statement that safety flaws in battery and pump connection to VAD controllers still exist, the study implies that further improvements should be made to increase the usability of LVAD systems in particular with regard to the controller interface. A simple modification would be to add markers to both controllers, providing a clear guidance in case of a controller change emergency. Dunn et al. [11] states resources how to further improve LVAD design for human factors. The next step for further research would be to evaluate and compare multiple LVAD devices regarding their design and user experience.

To improve the training effect a regular repetition (once a year) of an emergency controller change, is suggested. The results of our study underline the statement of Kormos et al. [10] that although non-pump related device malfunctions occur infrequently, it is highly relevant to carefully prepare patients, caregivers, allied medical personnel and physicians. This was confirmed by a study with paramedics, that came to the conclusion that LVAD device usability and training for emergencies must be improved because the LVAD handling is not intuitive enough [14]. Our study is the first one demonstrating the use of eye tracking for the assessment of the usability and handling of LVAD systems. Its use may be expanded to other critical medical devices in the in-hospital or ambulatory setting.

In conclusion, eye tracking was successfully used to quantitatively and objectively measure the patient’s performance in changing the battery or controller of a training HVAD. It is a valuable tool to control the success of the HVAD handling training. This training shows to be effective, but regular re-training of emergency scenarios will add safety. The gaze point during BC and CC should be analyzed by the eye tracking system during training and on regular basis during follow up to monitor a constant level of life important skills. Based on the results and conclusions of this study the institutional HVAD training routine has been improved to increase patients’ safety.

## Supplementary Information

Video 1 Eye tracking scan path video of HVAD battery and controller change for patients and experts (Video1_ScanPaths.mp4) (MP4 41,025 kb)
